# Colonization, mouse-style

**DOI:** 10.1186/1741-7007-8-131

**Published:** 2010-10-26

**Authors:** Sofia I Gabriel, Fríða Jóhannesdóttir, Eleanor P Jones, Jeremy B Searle

**Affiliations:** 1 CESAM - Centre for Environmental and Marine Studies, Departamento de Biologia Animal, Faculdade de Ciências da Universidade de Lisboa, 1749-016 Lisbon, Portugal; 2 Department of Ecology and Evolutionary Biology, Cornell University, Corson Hall, Ithaca, NY 14853-2701, USA; 3 Population Biology and Conservation Biology, Evolutionary Biology Centre, Uppsala University, Norbyvägen 18 D, SE-752 36 Uppsala, Sweden

## Abstract

Several recent papers, including one in *BMC Evolutionary Biology*, examine the colonization history of house mice. As well as background for the analysis of mouse adaptation, such studies offer a perspective on the history of movements of the humans that accidentally transported the mice.

See research article: http://www.biomedcentral.com/1471-2148/10/325

## Commentary

Commensals of humans are likely to share the human global distribution. Being easily noticed, and surviving and reproducing well in environments that humans create, they also include some of the most favored model organisms. A prime example of this is the house mouse, *Mus musculus*, which is both the 'classic' mammalian model organism and a globally present commensal. Through its association with humans, the house mouse is even found in the remotest archipelagos, such as Kerguelen, a group of sub-Antarctic islands with a mean summer temperature as low as 8°C. It is the mouse populations inhabiting this inhospitable place that are the focus of a study by Hardouin *et al*. in *BMC Evolutionary Biology*[1].

With all the genomic tools available, there is currently a scramble to study the genetics of adaptation in house mice. What better place to study that than Kerguelen? Here, human occupancy is restricted to the few inhabitants of a research station on the main island (Grande Terre). The mice on these islands live outdoors in extreme conditions and, in contrast to the typical seed-eating of house mice elsewhere, they feed primarily on invertebrates. To understand the adaptations for this exceptional lifestyle, it is important to know something about the history of the mice. In particular: where did the mice come from? Is it a genetically mixed population? Is the population young or old? Hardouin *et al*. [1] investigated all these questions for Kerguelen house mice which belong to the western subspecies, *Mus musculus domesticus*.

## The Kerguelen study

Hardouin *et al*.'s study [1] involved 437 mice from Kerguelen, an unprecedented coverage for the analysis of colonization history of such a small area. They found remarkable consistency in the mitochondrial DNA (mtDNA) sequences on Grande Terre and most of the surrounding small islands, suggesting that these populations are the product of a single relatively recent colonization (ultimately deriving from Europe). This fits with the recorded discovery of the archipelago in 1772 (by a Frenchman called Kerguelen-Trémarec) and settlement by mice either at the time or with subsequent human arrivals. Two of the other small islands in the archipelago (Cochons and Cimetière) may have been colonized in a second, separate introduction, as their mice belong to a different mtDNA lineage (also ultimately European). Over the archipelago as a whole there was no evidence of within-island heterogeneity in terms of mtDNA lineage. This is surprising given the large number of ships carrying mice that would have visited the islands (coming from many different places and therefore carrying mice of many different mtDNA lineages). These results are consistent with other data [2] suggesting that mouse populations are resistant to secondary invasion by females (mtDNA is a maternally inherited marker). Presumably, newly arriving females coming into an established population are generally unable to survive or gain mates, and in consequence do not contribute to the population's gene pool. All of this means that mtDNA may be a very good marker for initial colonization by house mice within a given area.

## Studies on European mice

**Figure 1:**
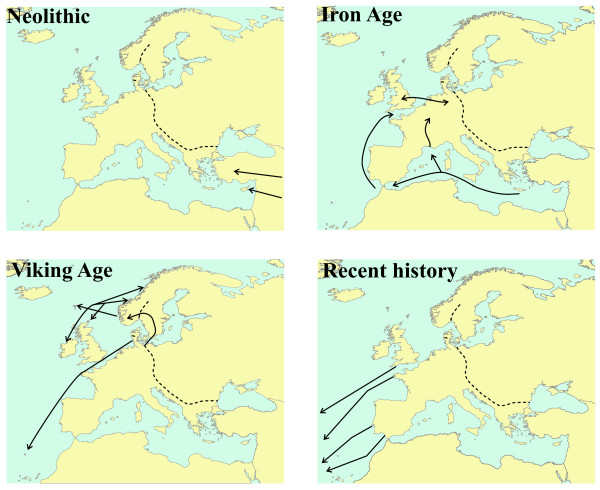
**Maps showing possible colonization routes taken by the western house mouse *Mus musculus domesticus*, based on mtDNA evidence**. Neolithic (starting 12,000 years ago): colonization restricted to the eastern Mediterranean close to where this subspecies first became commensal [3]. Iron Age (starting 3,000 years ago): colonization westwards along the Mediterranean and then into north-west Europe by overland and coastal routes [3,5]. Viking Age (around 1,000 years ago): movements around the periphery of north-west Europe and colonization of Scandinavia and Madeira [2,5,7,8]. (The colonization of Scandinavia may have been earlier [7].) Recent history (a few hundred years ago): mice were taken substantial distances from western Europe, including to Kerguelen [1]. The dashed line shows the location of the hybrid zone between the subspecies M. m. domesticus and M. m. musculus.

The association of *Mus musculus domesticus* with humans in a European context has long been studied by zooarcheologists, and genomic tools are now being deployed to study adaptation in some of these European populations. With regard to colonization history, there is much zooarcheological evidence on the progression of mice from their site of first commensalism with humans in the Near East through the Mediterranean region, providing a good test on the match between mtDNA sequences and the historical record. Gratifyingly, Bonhomme *et al*. [3] have identified a discontinuity in mtDNA lineages that fits very well with the two phases of mouse colonization of the Mediterranean revealed by zooarcheologists (Figure 1). The eastern Mediterranean was colonized by mice during the Neolithic when they were first able to exploit stored grain. However, the western Mediterranean could not be colonized by house mice until the Iron Age (Figure 1), when settlements reached a sufficient size for the house mice not to be outcompeted by local mice living outdoors, and when seafarers such as the Phoenicians carried cargoes of sufficiently large size to inadvertently transport house mice [3,4].

Studies by ourselves and others have looked at the mtDNA lineages of house mice in northern Europe. A different lineage from those typically seen in Mediterranean Europe has been found further north in the area between Britain and Germany [2,5]. The mouse mtDNA again matches a regional sphere of influence of Iron Age people [6], and, unlike other mouse mtDNA lineages, it appears that this Anglo-German lineage did not arrive in northern Europe by an overland route; instead it probably came along the Atlantic coast (Figure 1).

mtDNA studies suggest another pulse of detectable mouse colonizations during Viking times (Figure 1). Like the Phoenicians, the Vikings were impressive seafarers, carrying substantial cargoes ideal for stowaway mice, and there are mtDNA signals of maritime colonization events [2,5,7,8].

How did *Mus musculus domesticus* get from Europe to Kerguelen? This subspecies had been in the right place at the right time to make use of the first storage of grain by Neolithic humans in the Fertile Crescent in the Middle East, and to adapt to changing human cultural practices. Good fortune struck again when the subspecies found itself in western Europe at the time that British, Dutch, French and Iberian seafarers were 'discovering', exploiting and taking settlers to the rest of the world. Kerguelen-Trémarec and his crew may have been the first humans to see the archipelago that now bears his name, but the colonization route of the first mice to arrive there is still uncertain, although their starting point was certainly western Europe (Figure 1).

## Mice as a proxy for human history

It is intriguing how far the linkage between human history and mouse history may go. Jones *et al*. [8] found a correlation between mouse genetic diversity and human population size (proportional to amount of mouse habitat) in discrete areas of the Faroe Islands in the northeastern Atlantic Ocean, another archipelago where house mice have been studied. This supports the expectation that the population genetics (in terms of genetic response to population expansions and contractions) of house mice is likely to reflect rather closely the population genetics of humans.

We have been considering how the history of humans impacts on the genetics of the house mouse, but that can be turned around. If the history of house mice is so intimately determined by humans, then the genetics of house mice may be useful to answer human historical questions; for example, the details of human affiliations in the Iron Age are sometimes imprecise - might house mice be able to indicate associations between Iron Age people from different geographical areas? House mice are equivalent to an artifact that an archeologist discovers and uses to determine human colonization or trading routes. The provenance of the mice is established from their DNA sequence and that is a very powerful tool, given its extraordinary information content. Not only can the DNA sequence help to establish the source of the mice found in a particular place but it can be used to date the original colonization and subsequent population history (including secondary colonizations), following approaches used for human DNA (see, for example [9])). However, it is clear from all the recent papers considered here [1,3,5,7,8] that archeogenetics using house mice is at an early stage, and that, in particular, calibrations to generate an accurate mouse mtDNA molecular clock are urgently needed. Hardouin *et al*. [1] comment that, for the mtDNA region analyzed, they found a much higher mutation rate than suggested by previous studies. Further work should follow up this finding and also use other subspecies to globalize the opportunities for applying mice as a proxy to study humans, following the lead of another recent paper [10].
